# Novel missense variants in the *RNF213* gene from a European family with Moyamoya disease

**DOI:** 10.1038/s41439-019-0066-6

**Published:** 2019-08-08

**Authors:** Andrey N. Gagunashvili, Louise Ocaka, Daniel Kelberman, Pinki Munot, Chiara Bacchelli, Philip L. Beales, Vijeya Ganesan

**Affiliations:** 1 GOSgene, Genetics and Genomic Medicine, UCL Great Ormond Street Institute of Child Health, London, UK; 2 Neurology Department, Great Ormond Street Hospital for Children NHS Foundation Trust, London, UK; 3 Clinical Neurosciences, UCL Great Ormond Street Institute of Child Health, London, UK

**Keywords:** Stroke, Genetics research

## Abstract

In this report, we present a European family with six individuals affected with Moyamoya disease (MMD). We detected two novel missense variants in the Moyamoya susceptibility gene *RNF213*, c.12553A>G (p.(Lys4185Glu)) and c.12562G>A (p.(Ala4188Thr)). Cosegregation of the variants with MMD, as well as a previous report of a variant affecting the same amino acid residue in unrelated MMD patients, supports the role of *RNF213* in the pathogenesis of MMD.

Moyamoya (MM) is a rare cerebrovascular disorder that is characterized by a high rate of stroke and frequent recurrence. When MM is associated with a recognized genetic or acquired condition, it is termed MM syndrome (MMS). The majority of cases appear to be idiopathic in origin and are termed MM disease (MMD). Linkage studies have identified a number of loci in reports of cases, albeit with incomplete penetrance, suggesting a role for genetic factors in the etiology of MMD. Elucidation of the genes involved has been hampered by several complicating factors, including clinical and radiological phenotypic heterogeneity, incomplete penetrance and variable onset of the disease. The inheritance pattern in the majority of cases is unclear. The use of whole-exome sequencing has allowed for the identification of a number of rare variants in genes linked to rare Mendelian forms, particularly when associated with a broader spectrum of disease in patients with MMS. However, in idiopathic cases of MMD, variations in the *RNF213* gene have been identified as conferring susceptibility to MMD. In particular, a founder variant c.14429G>A (p.(Arg4810Lys) (dbSNP accession rs112735431)) has been observed in 80% of Japanese and East Asian MMD patients, compared to 2% in the general population^1,2^. The diversity of susceptibility variants in MMD patients of European ancestry remains underrepresented.

In this report, we analyzed a white European family from the UK with six relatives affected with MMD over three generations (Fig. 1a) (for a detailed description of the clinical presentation, see Supplementary Text S1). In an attempt to find a genetic cause of MMD in this family, two affected and three unaffected members of the family (denoted with a plus sign in Fig. 1a) underwent whole-exome sequencing to an average coverage of 104.6× (range of 98.4–108.5×) such that, on average, 96.2% of the exome capture target regions was covered by at least 20× (for a detailed description of the methods see Supplementary Text S2). We focused our analysis on coding and splice region variants (single nucleotide variants and indels) that are not present or rare (allele frequency <0.5%) in public databases of human genetic variation: ExAC/gnomAD, 1000 Genomes Project and NHLBI ESP.

**Fig. 1:**
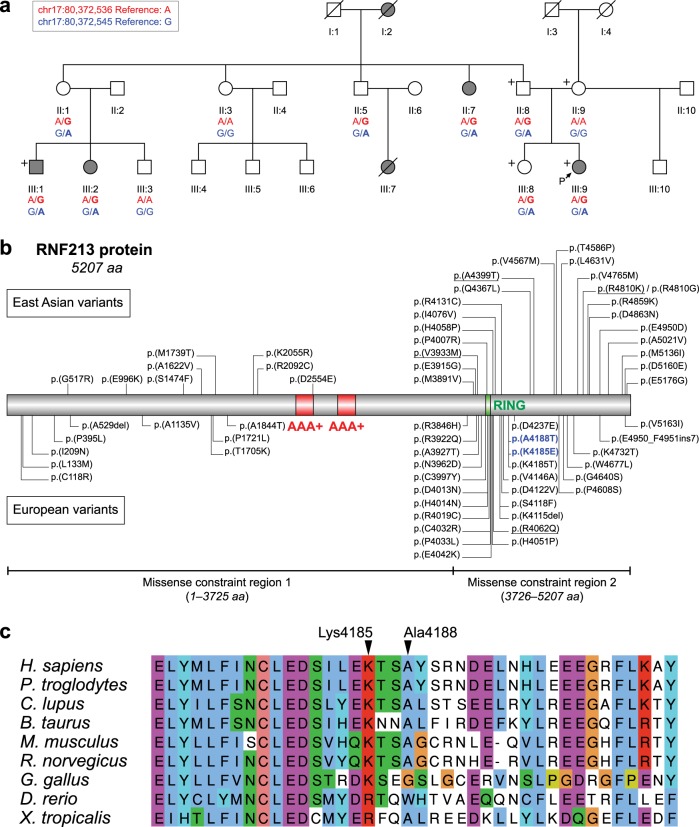
Novel missense variants in the *RNF213* gene from a European family with MMD. **a** Pedigree of the family. The genotypes of the two *RNF213* variants are shown below each family member for which a DNA sample was available, where red refers to the status of the missense variant c.12553A>G (p.(Lys4185Glu)), and blue refers to the status of the missense variant c.12562G>A (p.(Ala4188Thr)) (reference allele/alternate allele). Filled symbols denote affected individuals, unfilled symbols denote unaffected individuals, and slashed lines denote deceased. Five individuals who were whole-exome sequenced are denoted with a plus sign. P, proband. The genotypes of other family members were obtained with Sanger sequencing. **b** The domain structure of the RNF213 protein based on^14^ and variants previously reported in East Asian and European MMD patients^1,2,5,11–13,15–20^ (shown above and below the protein, respectively). The variants reported for both populations are underlined, and the ones identified in this study are shown in blue. Two distinct regions with different missense constraints identified by Samocha and coworkers^6^ are shown below the protein. **c** Conservation of amino acid residues affected by the c.12553A>G (p.(Lys4185Glu)) and c.12562G>A (p.(Ala4188Thr)) variants. AAA+, ATPases associated with various cellular activity domains; RING, RING-finger domain

Analysis of filtered genetic variants was performed assuming a dominant mode of inheritance. X-linkage could not be excluded due to the absence of any male-to-male transmission within the pedigree. However, there is at least one instance of nonpenetrance in the family as individual II:8, although clinically unaffected, is an obligate carrier of the variant(s) that he passed onto his affected daughter (III:9). Consequently, individual II:8 was classified as “affected” for the purpose of the genetic analysis (for a detailed description of the variant analysis, see Supplementary Text S3). The observation of incomplete penetrance is consistent with other reports of MMD inheritance^3,4^.

No potential candidate variants were identified that were consistent with an X-linked dominant mode of inheritance (Supplementary Fig. S2). Analysis of the autosomes identified 20 candidate variants, 16 of which were predicted to be deleterious to protein function (missense and frameshift variants) (Supplementary Fig. S2, Supplementary Table S1). Of these sixteen candidates, two missense variants, c.12553A>G (p.(Lys4185Glu)) and c.12562G>A (p.(Ala4188Thr)) (Supplementary Table S1), were identified in the *RNF213* gene, which has previously been reported as a major susceptibility gene for MMD^1,2^. No other candidates were identified in our analysis that could explain the disease in the affected family members.

The parental aunt (II:1) and uncle (II:5) of the proband are both obligate carriers of these variants, both having affected children (III:1, III:2 and III:7) (Supplementary Fig. S3 and S4). The clinically unaffected sister of the proband (III:8) also harbors the variants, further demonstrating the incomplete penetrance characteristic of the condition (Supplementary Fig. S3 and S4, Supplementary Table S2). These individuals are clinically asymptomatic and are currently undergoing clinical evaluation. Brain imaging studies have not been undertaken to date.

The variants identified are currently private to the family and have not been previously documented or reported in public databases (Table 1). Both variants cosegregate with MMD in multiple affected family members and obligate carriers (Supplementary Fig. S5). The p.(Lys4185Glu) is a conservative amino acid substitution that occurs at a position that is conserved across species (Fig. 1c, Supplementary Table S3). Multiple lines of computational evidence support a deleterious effect of the c.12553A>G (p.(Lys4185Glu)) variant on the gene product (Supplementary Table S3). A heterozygous missense variant affecting the same lysine residue (c.12554A>C, p.(Lys4185Thr)) has also been previously reported in an unrelated family of European ancestry with MMD^5^. The c.12554A>C variant also has similar deleterious in silico predictions to those for p.(Lys4185Glu) (Supplementary Table S3). Predictions for the c.12562G>A (p.(Ala4188Thr)) variant were mostly benign, suggesting that this variant may have a limited impact on the resultant protein (Supplementary Table S3).

**Table 1:** Summary of the *RNF213* variants found in the European family with MMD

	Variant 1	Variant 2
Position (GRCh38)	chr17:80,372,536	chr17:80,372,545
Variant consequences	Missense	Missense
Variant genotype	Heterozygous	Heterozygous
cDNA change	c.12553A>G (NM_001256071.2)	c.12562G>A (NM_001256071.2)
Protein change	p.(Lys4185Glu) (NP_001243000.2)	p.(Ala4188Thr) (NP_001243000.2)
Transcript length	21,062 bp (NM_001256071.2)	21,062 bp (NM_001256071.2)
Protein length	5,207 aa (NP_001243000.2)	5,207 aa (NP_001243000.2)
Exon/exons in transcript	48/68 (NM_001256071.2)	48/68 (NM_001256071.2)
Allele frequency in public databases:		
ExAC/gnomAD	0	0
1000 Genomes	0	0
NHLBI ESP	0	0
Presence in dbSNP	Not present	Not present
Variant classification	Likely pathogenic	Uncertain significance
ClinVar accession	SCV000839587	SCV000839588

Genetic studies of familial MMD are complicated by considerable heterogeneity, incomplete penetrance of the condition and variable onset of the disease. *RNF213* has been identified as a major susceptibility gene for MMD, mostly in cases of East Asian ancestry. Interestingly, both East Asian and European MMD patients show very little overlap in *RNF213* variants associated with the disease (Fig. 1b). Apart from the founder variant c.14429G>A (p.(Arg4810Lys))^2^, the differences between East Asian and European MMD-associated variants can be explained by a spread of spontaneously arisen mutations.

According to the gnomAD database, *RNF213* is an unconstrained gene with a high tolerance to both missense (2527 observed vs. 2920 expected, z-score: 2.64) and loss-of-function (124 observed vs. 221.8 expected, pLI-score: 0) variants. Nevertheless, there are no homozygous loss-of-function *RNF213* variants in gnomAD (accessed on January 2019), suggesting that complete loss of the *RNF213* gene would not be tolerated. In contrast, highly constrained genes harboring variants that result in haploinsufficiency are known to cause severe disease^6^. This observation can explain the reduced penetrance of MMD in families carrying rare *RNF213* variants and suggests that other factors, including genetic background, are required to trigger the development of the disease.

In this case, we identified two novel missense variants in the *RNF213* gene in a European family with MMD. The occurrence of two private variants on the same allele 7 bp apart (Supporting Fig. S3) suggests the possibility that they have arisen as a result of a single mutational event^7^. It has been shown previously that such clustered, multinucleotide mutations can be introduced by the normal activity of the more error-prone components of the DNA repair pathway, such as polymerase zeta^8^. The presence of an indel or a larger structural variant at the locus was not evident from the mapped sequencing reads (Supplementary Fig. S3).

The variants are situated in the C-terminal region of the RNF213 protein, where the majority of MMD-associated variants have been reported (Fig. 1b). This region harbors a domain for a RING-finger E3 ligase, which is involved in the ubiquitination of substrates targeted for either proteasomal degradation or signal transduction. Interestingly, this part of RNF213 represents one of the two distinct segments (1–3725 and 3726–5207 amino acids) with different missense constraints identified by Samocha and coworkers^6^ (Fig. 1b). This suggests that missense variants in this region may have different deleterious effects on the protein function compared to the variants in the first segment of RNF213. Applying the American College of Medical Genetics and Genomics and the Association for Molecular Pathology (ACMG/AMP) guidelines^9,10^, we interpreted the p.(Lys4185Glu) variant in the *RNF213* gene as likely pathogenic for MMD, acting in a dominant manner. The p.(Ala4188Thr) variant was interpreted as a variant of uncertain significance with respect to MMD due to conflicting/insufficient evidence (for a detailed description of the variant interpretation see Supplementary Text S4). We could not exclude the possibility of a synergistic effect of having both of these variants in cis to cause the phenotype. It will be interesting whether other unrelated MMD patients who carry only one variant will be found. The study of such cases may not only confirm the role of the variants in the pathogenesis of MMD but may also reveal some phenotypic differences between the carriers. The multiple reported occurrence of individuals exhibiting reduced penetrance for a particular disease can complicate genetic studies and make it difficult to draw firm conclusions regarding the association of a particular gene with disease pathogenesis. To overcome this, the reporting of novel variants associated with diseases such as MMD is important to greatly facilitate variant interpretation in the future. Multiple recurrences of the same variant or variants within the same gene (particularly affecting the same amino acid residue) in unrelated patients provide increasing supportive evidence for disease association. Our findings support and add more evidence to the importance of alteration of the C-terminal region of RNF213, including the RING-finger domain, in the pathogenesis of MMD. This study also adds important weight to a growing body of evidence^11–13^ that variation in the *RNF213* gene also has an important role in disease susceptibility in European populations.

## Supplementary information

Supplementary material - Novel missense variants in the RNF213 gene from a European family with Moyamoya disease.

## Data Availability

The relevant data from this Data Report are hosted at the Human Genome Variation Database at 10.6084/m9.figshare.hgv.2588, 10.6084/m9.figshare.hgv.2591.

## Data Availability

The variants identified in this study were deposited in the ClinVar database under accessions SCV000839587 and SCV000839588. Additional data can be found in the Supplementary Information online.
