# Supplementary material for: Novel missense variants in the RNF213 gene from a European family with Moyamoya disease
Source: Hum Genome Var. 2019 Aug 8;6:35. doi: 10.1038/s41439-019-0066-6 (PMC6804521; doi:10.1038/s41439-019-0066-6)
Supplement: Supplementary file 1 — Supplementary material - Novel missense variants in the RNF213 gene from a European family with Moyamoya disease. [file 41439_2019_66_MOESM1_ESM.pdf]

# Novel missense variants in the *RNF213* gene from a European family with Moyamoya disease

Andrey Gagunashvili[1], Louise Ocaka[1], Dan Kelberman[1], Pinki Munot[2]
Chiara Bacchelli[1], Philip L. Beales[1] & Vijeya Ganesan[2,3]

## Supplementary Information

[1] *GOSgene, Genetics and Genomic Medicine, UCL Great Ormond Street Institute of Child Health, London, United Kingdom*
[2] *Neurology Department, Great Ormond Street Hospital for Children NHS Foundation Trust, London, United Kingdom*
[3] *Clinical Neurosciences, UCL Great Ormond Street Institute of Child Health, London, United Kingdom*

Table of Contents

**Supplementary text S1** Clinical presentation . . . . . 3
**Supplementary text S2** Methods . . . . . 4
**Supplementary text S3** Variant analysis . . . . . 6
**Supplementary text S4** Variant interpretation . . . . . 7
**Figure S1** Catheter cerebral angiograms of the proband . . . . . 8
**Figure S2** Variant filtering flowchart . . . . . 9
**Figure S3** Visualisation of exome sequencing results in the Integrative Genomics Viewer . . . . . 10
**Figure S4** Sanger sequencing validation of *RNF213* variants in the family . . . . . 11
**Figure S5** Cosegregation of the *RNF213* variants with MMD in the family . . . . . 12
**Figure S6** Interpretation of the *RNF213* variants according to the ACMG/AMP guidelines . . . . . 13
**Table S1** Rare deleterious exonic variants with required genotypes . . . . . 15
**Table S2** Sequencing data metrics for the *RNF213* missense variants . . . . . 16
**Table S3** dbNSFP predictions for the *RNF213* missense variants . . . . . 17
**References** . . . . . 18

# Supplementary Text S1.

## Clinical presentation

The female proband (III:9, Figure S5) presented at the age of 4 years with two episodes of transient left sided weakness and aphasia, brought on by excitement. Both episodes resolved completely. She also had a long-standing history of alternate day headache, without other associated features. She underwent brain magnetic resonance imaging, and subsequently cerebral angiography (Supplementary Figure S1) that confirmed the diagnosis of bilateral Moyamoya disease (MMD). She was commenced on aspirin, underwent right sided pial synangiosis and has subsequently remained clinically and radiologically stable for over 5 years. Of note, there was a positive family history of MMD in at least 2 generations on her paternal side (Figure S5). Her paternal grandmother (I:2) was diagnosed with MMD and died aged 65 years. Her father (II:8) had brain imaging for investigation of epilepsy that did not show evidence of MMD. Her paternal aunt (II:1) had 3 children; she was unaffected as was her youngest son (III:3). However her other 2 children (III:1 and III:2) had a diagnosis of MMD, having presented with stroke aged 5 and a movement disorder, respectively. The paternal uncle (II:5, clinically unaffected) had a child who died from a brain haemorrhage aged 7 years although no formal diagnosis of MMD is recorded. Another paternal aunt (II:7) has a diagnosis of MMD, having suffered a stroke at the age of 21. Finally the other paternal aunt, (II:3) had 3 sons. One of the sons (III:4) was under investigation for learning difficulties, without current diagnosis, and the remaining sons (III:5 and III:6) were in good health.

## Supplementary Text S2.

### Methods

**Diagnosis of Moyamoya disease:** Diagnosic criteria of MMD by the Research Committee on the Pathology and Treatment of Spontaneous Occlusion of the Circle of Willis (Moyamoya disease) in Japan (Fukui, 1997; Hashimoto et al., 2012) were used for diagnosis of MMD in the affected members of the family and include all of the following items based on the conventional angiographic findings: (i) stenosis or occlusion of the terminal portion of the intracranial internal carotid artery or proximal portions of the anterior cerebral artery and/or the middle cerebral artery, (ii) development of abnormal vascular networks near the occlusive or stenotic lesions in the arterial phase, (iii) bilaterality of findings (i) and (ii).

**Whole exome sequencing and alignment:** Whole exome capture and sequencing was performed at BGI (Shenzhen, China) using SureSelect Human All Exon v4 51 Mb kit (Agilent Technologies, Santa Clara, CA, USA) and Illumina HiSeq2000 System (Illumina, San Diego, CA, USA). We aimed for a mean coverage of 100× for the exome capture target regions. Sequencing reads were aligned with Burrows-Wheeler Aligner (BWA) v0.7.17 (Li and Durbin, 2010) to human genome build 38 (GRCh38.p1) not including alternate assemblies (GCA_000001405.15_GRCh38_no_alt_analysis_set.fna) and read duplicates were marked with Sambamba (Tarasov et al., 2015).

**Variant calling and annotation:** Variant calling across the exome capture target regions with 100 bp padding was performed using Genome Analysis Toolkit (GATK) v4.0.3.0 (DePristo et al., 2011; McKenna et al., 2010) according to the best practices workflow for joint (multi-sample) calling (Van der Auwera et al., 2013). The result variants were normalized and decomposed using Bcftools v1.8 (*https://github.com/samtools/bcftools*) and annotated with ANNOVAR (Wang et al., 2010) and dbNSFP v4.0b1 (Liu et al., 2011; Dong et al., 2014; Liu et al., 2016).

**Variant filtering:** Rare variants were defined as those having an allele frequency lower than 0.5% in public databases: ExAC (v0.3.1) (Lek et al., 2016), gnomAD (v2.0.2) (Lek et al., 2016), 1000 Genomes (phase 3) (1000 Genomes Project Consortium et al., 2015) and NHLBI ESP (ESP6500SI-V2; *evs.gs.washington.edu/EVS/*). Genotype requirements for autosomal dominant mode of inheritance were following: (i) all affecteds must be heterozygous (0/1), (ii) no unaffected can be heterozygous (0/1) or homozygous alternate (1/1), (iii) if there is incomplete penetrance suspected in the kindred with unaffected obligate carriers, these individuals must be considered affected (0/1). Genotype requirements for X-linked dominant mode of inheritance were following: (i) affected males are heterozygous (0/1) or homozygous alternate (1/1), (ii) affected females must be heterozygous (0/1), (iii) unaffecteds must be homozygous reference (0/0).

**Variant interpretation:** The modified criteria from the American College of Medical Genetics and Genomics and the Association for Molecular Pathology (ACMG/AMP) guidelines were used for variant classification (Richards et al., 2015; Gelb et al., 2018). Pathogenicity and benign evidence tags was input into ClinGen Pathogenicity Calculator (Patel et al., 2017) for assessing pathogenicity of genetic variants.

**Sanger sequencing:** PCR amplicon sequencing was used to verify the presence of each mutation and to perform segregation analysis. The target region was amplified using primers (forward 5′-TCCACAGTCACATTGGCGACT-3′ and reverse 5′-AGCCCAGTAGTAGTCTATTCG-3′). PCR products were purified with ExoSap and sequenced using Big Dye Terminator Cycle Sequencing Kit v3.1 (Life Technologies, Foster City, CA, USA). Result electropherograms were analyzed with Geneious software (Biomatters Ltd., Auckland, New Zealand).

## Supplementary Text S3.

### Variant analysis

To identify a causative variant in the family affected with MMD over three generations, we performed whole exome sequencing of five family members: II:8 (father), II:9 (mother), III:1 (cousin), III:8 (sister) and III:9 (proband). We focused our analysis on rare (allele frequency <0.5% or absent in public databases) variants (SNVs and indels) within RefSeqGene exonic and splice regions, as annotated by ANNOVAR (Wang et al., 2010).

We promptly excluded the possibility of MMD being caused in this family by *de novo* mutations because its occurence in three generations. We also ruled out the possibility of autosomal recessive mode of inheritance because parental consanguinity was absent in the family and MMD occurred in three first cousins from two different marriages. X-linked recessive, Y-linked and mitochondrial modes of inheritance were excluded as well because both sexes were affected (with more affected females than males) and the disease appeared to be transmitted by either sex, including male-to-female. The most compatible mode of inheritance was found to be either X-linked dominant (XLD) or autosomal dominant (AD) since both sexes in subsequenct generations were affected with the same disease.

Because the pedigree was not fully consistent with AD or XLD mode, we suspected incomplete penetrance of the condition, supported by previous studies on MMD inheritance (Mineharu et al., 2006, 2008). Since the affected proband (III:9) most likely inherited the condition from her father (II:8) with a family history of MMD, we assumed that the father was an asymptomatic carrier of a dominant defective allele. Moreover, although the father did not have MMD *per se*, he had epilepsy which can be one of the clinical presentations of MMD (Cho and Tominaga, 2010; Koizumi et al., 2017). We applied the same assumption to the unaffected sister (III:8) that could have either 0/0 or 0/1 genotype. Assuming AD or XLD mode of inheritance with incomplete penetrance, the unaffected mother (II:9) was the only individual not related to the index case (I:2) and served as a control (genotype 0/0), while the father (II:8), the affected cousin (III:1), the sister (III:8) and the affected proband (III:9) were all case samples.

No variants following XLD mode of inheritance were identified (Figure S2). The search for the causative genotype under AD mode of inheritance in the affected family members resulted in 20 variants, with 16 of them being deleterious (missense and frameshift variants) (Figure S2, Table S1). Out of the sixteen variants, two missense variants (Table S1) were detected in the *RNF213* gene, previously associated with MMD (Kamada et al., 2011; Liu et al., 2011). Both variants were private to the family (with no presence in public databases) and were also found in the unaffected sister (III:8) (Figure S3, Table S2) supporting incomplete penetrance of the condition. Beside the *RNF213* variants, no other candidates that could explain the clinical phenotype were identified in our analysis.

## Supplementary Text S4.

### Variant interpretation

The c.12553A>G (p.(Lys4185Glu)) and c.12562G>A (p.(Ala4185Thr)) variants in affected family members and obligate carriers were observed in the *RNF213* gene, which has previously been reported as a major susceptibility gene for MMD (**PP4-Supporting**) (Kamada et al., 2011; Liu et al., 2011; Koizumi et al., 2017), and have not been published to our knowledge. Both variants were absent from NHLBI Exome Sequencing Project (*evs.gs.washington.edu/EVS/*), 1000 Genomes Project (1000 Genomes Project Consortium et al., 2015), or ExAC/gnomAD (Lek et al., 2016) databases (**PM2-Moderate**) and were private to the family. The *RNF213* variants cosegregate with MMD in multiple affected family members (**PP1-Supporting**) and obligate carriers. Since there are 5 segregations in the family (Supplementary Figure S5), we upgraded **PP1** evidence from default supporting to moderate strength (**PP1-Moderate**) according to the modified ACMG/AMP criteria for RASopathies (Gelb et al., 2018). Multiple lines of computational evidence support a deleterious effect of the c.12553A>G variant on the gene product (**PP3-Supporting**) (Supplementary Table S6). A heterozygous missense variant affecting the same lysine residue (c.12554A>C, p.(Lys4185Thr)) has been previously reported for another family of European ancestry with MMD (Smith et al., 2014; The Human Gene Mutation Database (HGMD) accession CM1414304). The p.(Lys4185Thr) variant has in silico predictions similar to those for p.(Lys4185Glu) (Supplementary Table S6) and is listed as "disease causing" in the HGMD database although no classification according to the ACMG/AMP guidelines is provided. Given that the p.(Lys4185Thr) variant does not meet "likely pathogenic"/"pathogenic" criteria, we downgraded **PM5** from default moderate to supporting strength (**PM5-Supporting**). Since there are 2 moderate and $\geqslant$2 supporting pathogenicity evidence tags, p.(Lys4185Glu) variant in the *RNF213* gene is therefore interpreted to be likely pathogenic for MMD and acts in a dominant manner (Supplementary Figure S6).

For the p.(Ala4185Thr) variant multiple lines of computational evidence suggest limited impact on gene product (**BP4-Supporting**) (Supplementary Table S6). We interpret p.(Ala4188Thr) variant in the *RNF213* gene as a variant of uncertain significance with respect to MMD due to conflicting/insufficient evidence ($\geqslant$1 supporting benign evidence tag, $\geqslant$1 moderate and $\geqslant$1 supporting pathogenicity evidence tags) (Supplementary Figure S6).

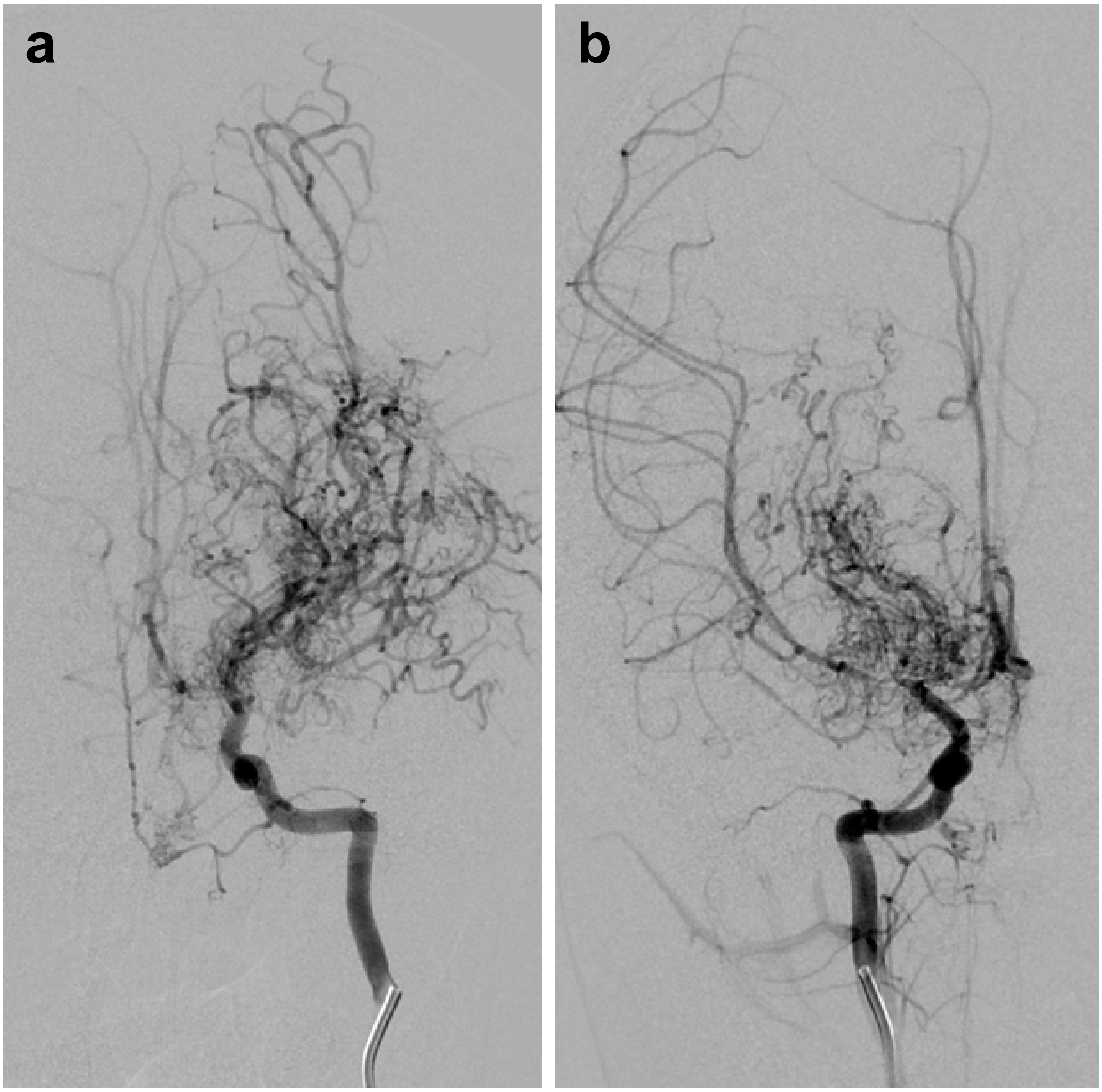


**Figure S1.** Catheter cerebral angiograms (frontal projection) of left (**a**) and right (**b**) internal carotid arteries showing severe occlusive disease of both middle and anterior cerebral arteries and typical basal "moyamoya" collaterals in the proband undertaken at the age of 6 years.

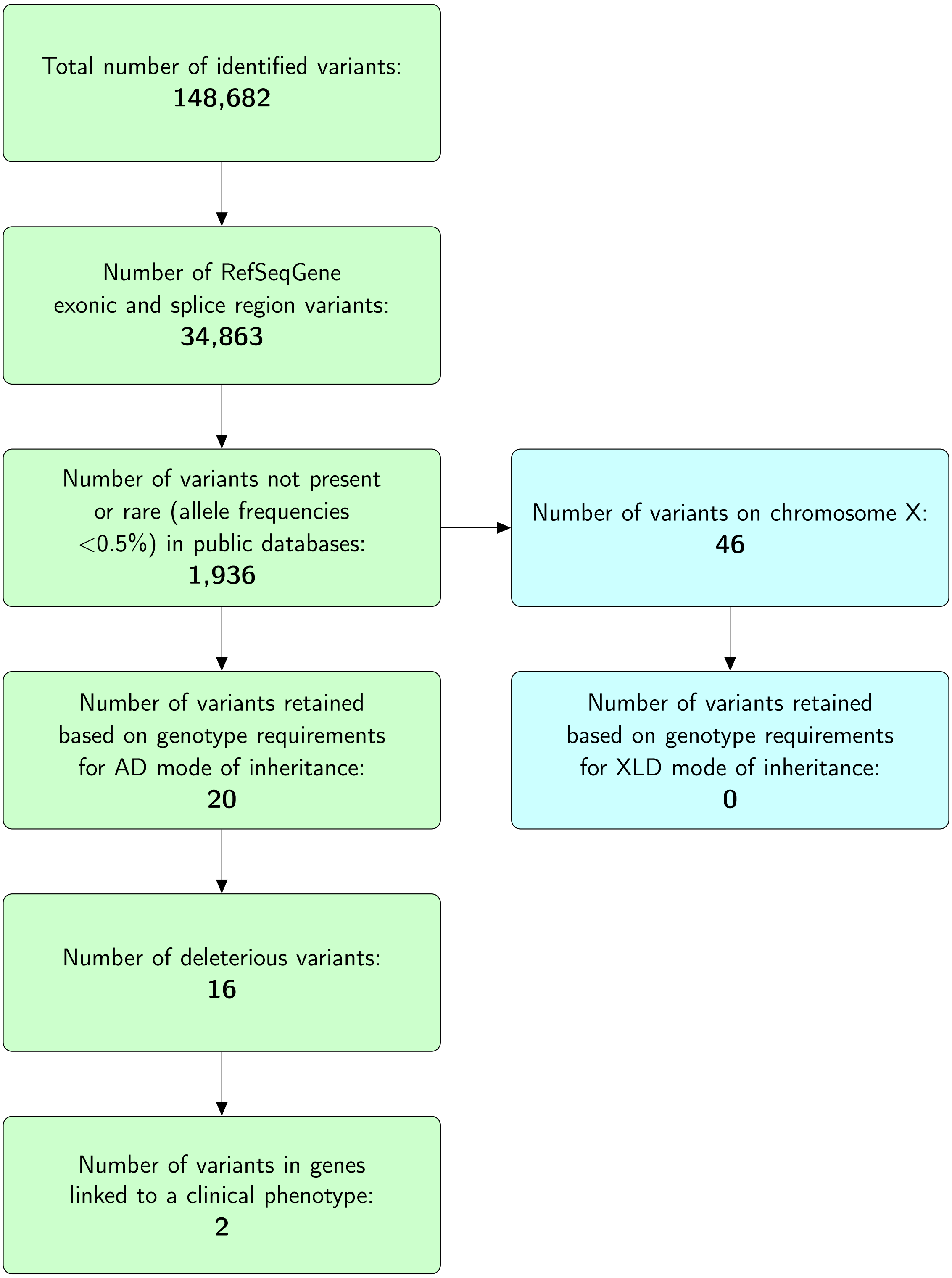


**Figure S2.** Variant filtering flowchart. In total 148,682 variants were identified in joint (multi-sample) calling for the sequenced family members. 34,863 variants lied in RefSeqGene exonic and splice regions. Out of these variants, 1,936 were not present or were rare (allele frequencies <0.5%) in public databases. Twenty variants were further retained based on genotype requirements for autosomal dominant (AD) mode of inheritance (see Supplementary Text S1), with 16 variants being deleterious (missense and frameshift variants). Out of the sixteen variants, two were found in the gene (*RNF213*) previously associated with the disease. No variants following X-linked dominant (XLD) mode of inheritance were identified.

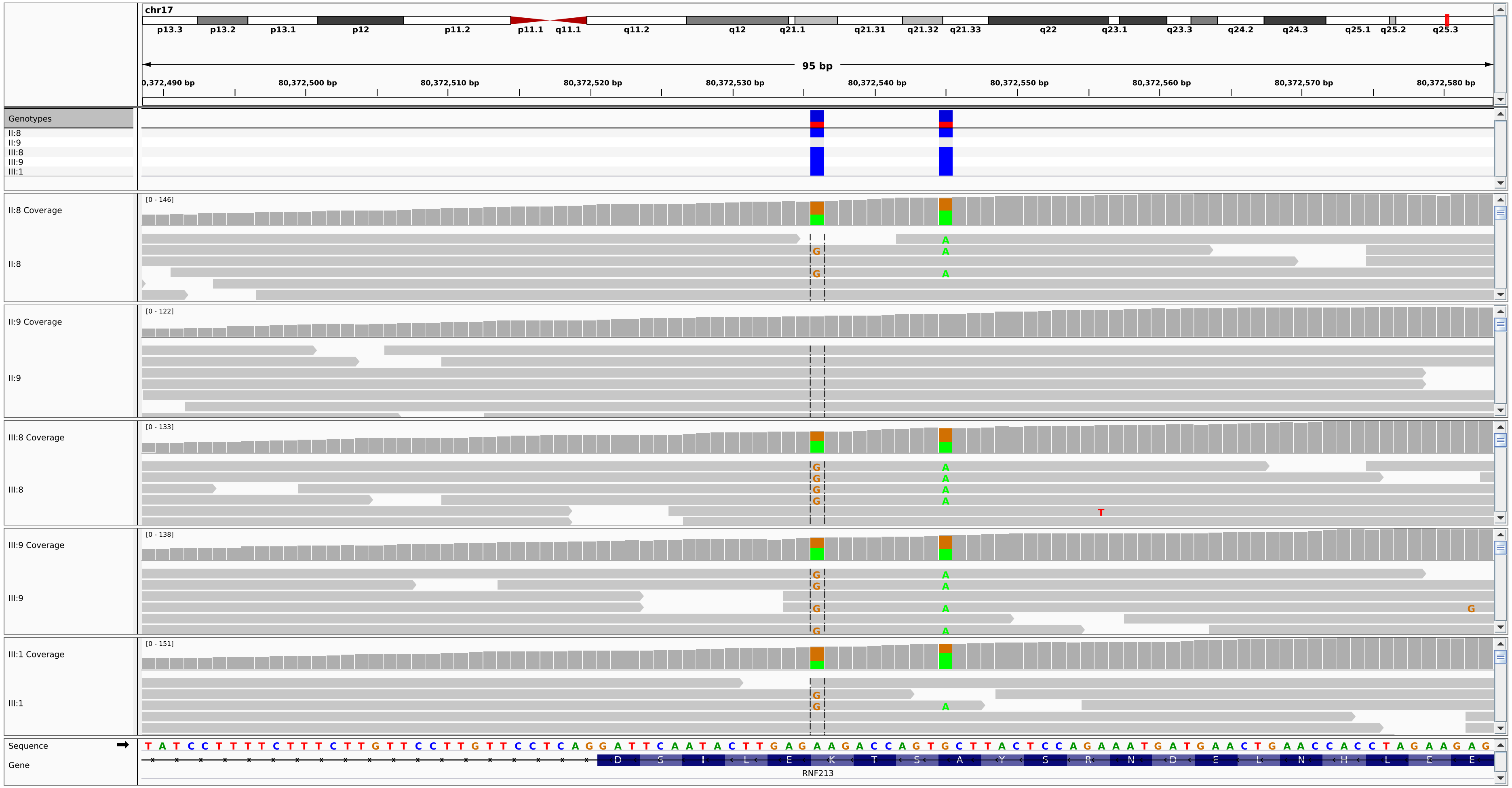


**Figure S3.** Visualisation of exome sequencing results in the Integrative Genomics Viewer (IGV) demonstrates identified heterozygous *RNF213* variants, c.12553A>G (p.(Lys4185Glu)) and c.12562G>A (p.(Ala4188Thr)), in the father (II:8), unaffected sister (III:8), proband (III:9) and affected cousin (III:1). Location of both variants on a same sequencing read indicates that they are located on the same chromosome.

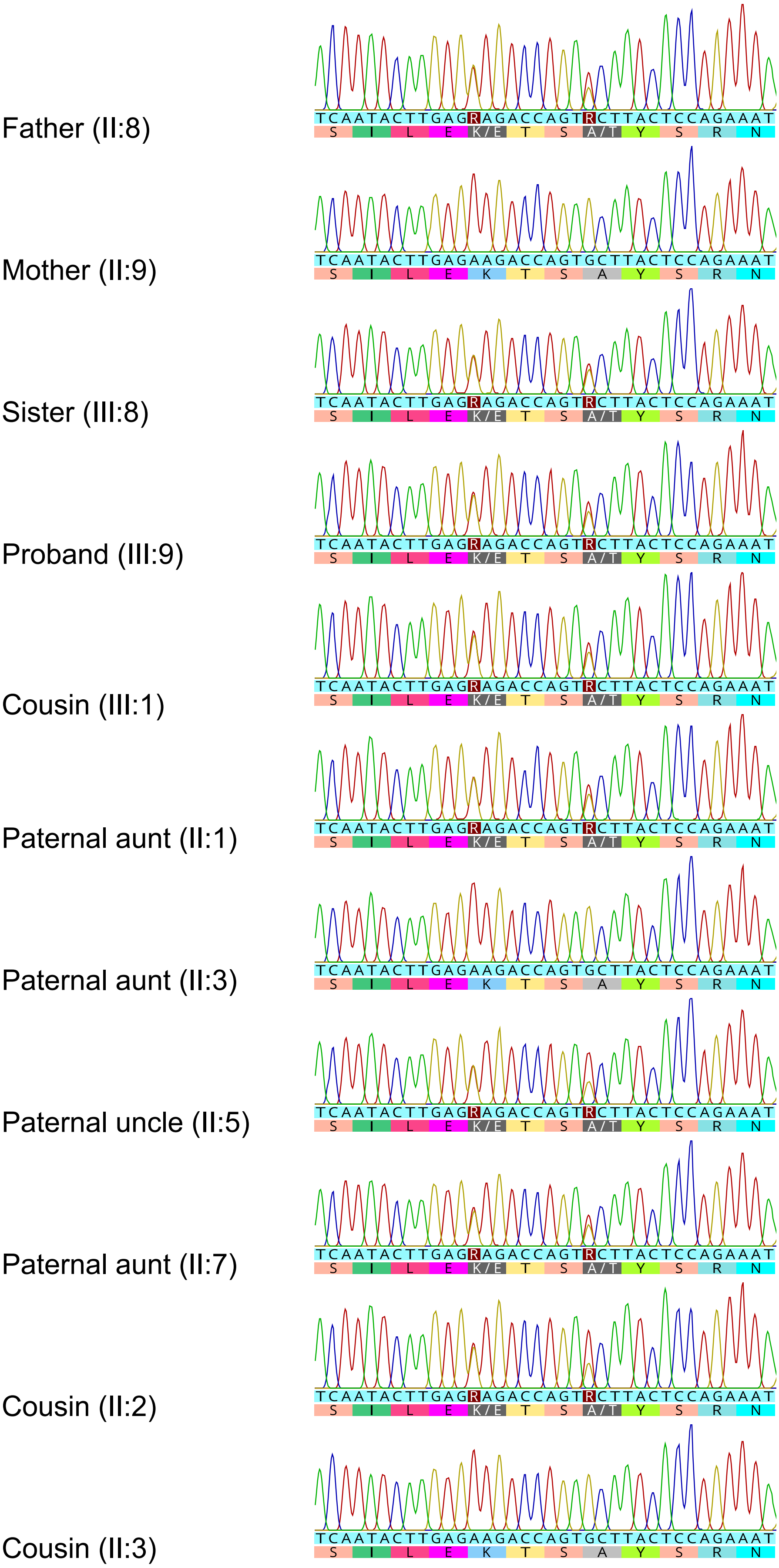


**Figure S4.** Sanger sequencing validation of *RNF213* variants, c.12553A>G (p.(Lys4185Glu)) and c.12562G>A (p.(Ala4188Thr)), in the family.

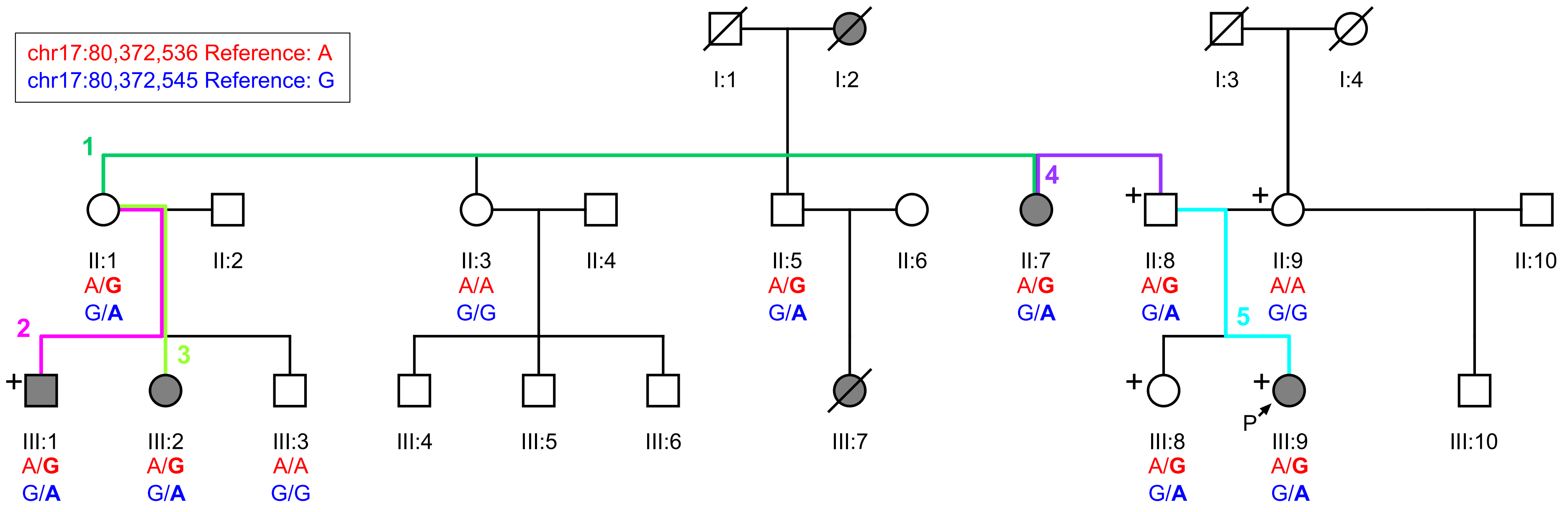


**Figure S5.** Cosegregation of the *RNF213* variants with MMD in the family. Segregations are shown as colored lines. The genotypes of the two *RNF213* variants are shown below each family member for which a DNA sample was available, where red refers the status of the missense variant c.12553A>G (p.(Lys4185Glu)), and blue refers to the status of the missense variant c.12562G>A (p.(Ala4188Thr)) (reference allele/alternate allele). Filled symbols denote affected individuals, unfilled symbols denote unaffected individuals, and slashed lines denotes deceased. Five individuals with whole exome sequenced are denoted with a plus sign. P, proband. The genotypes of other family members were obtained with Sanger sequencing. There are 2 segregations from individual II:7 to II:1 (obligate carrier) and II:8 (obligate carrier), 2 segregations from II:1 to III:1 and III:2 and 1 segregation from II:8 to III:9. Individuals I:2 and III:7 were affected but were not genotyped, so it was not counted as a segregation. Individual III:8 is genotype positive but is not affected probably due to a delayed onset of the disease and was not counted as a segregation either.

### Variant NM_001256071.2:c.12553A>G, NP_001243000.2:p.(Lys4185Glu)

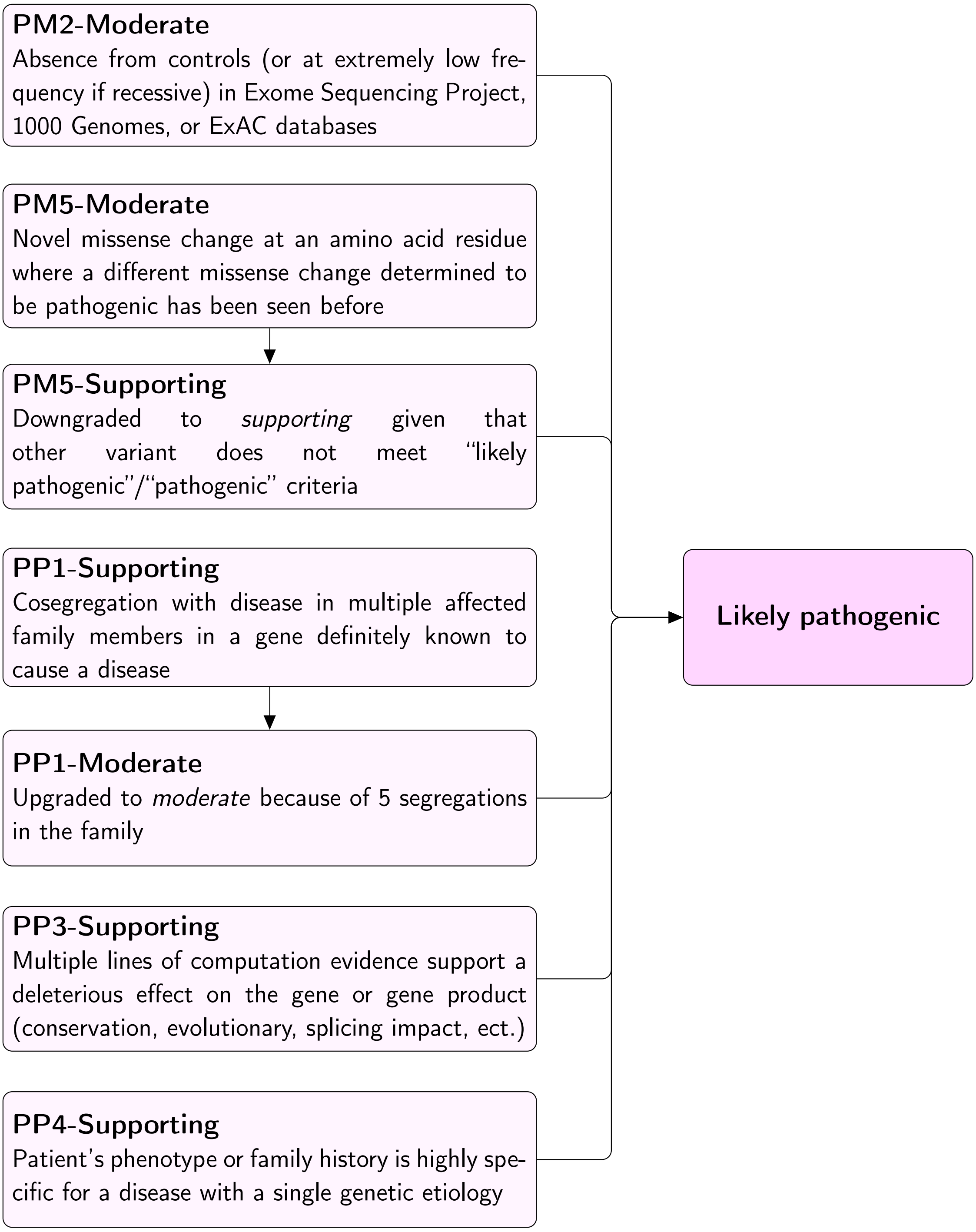


**Figure S6.** Interpretation of the *RNF213* variants according to the ACMG/AMP guidelines (Richards et al., 2015; Gelb et al., 2018).

Variant NM_001256071.2:c.12562G>A, NP_001243000.2:p.(Ala4188Thr)

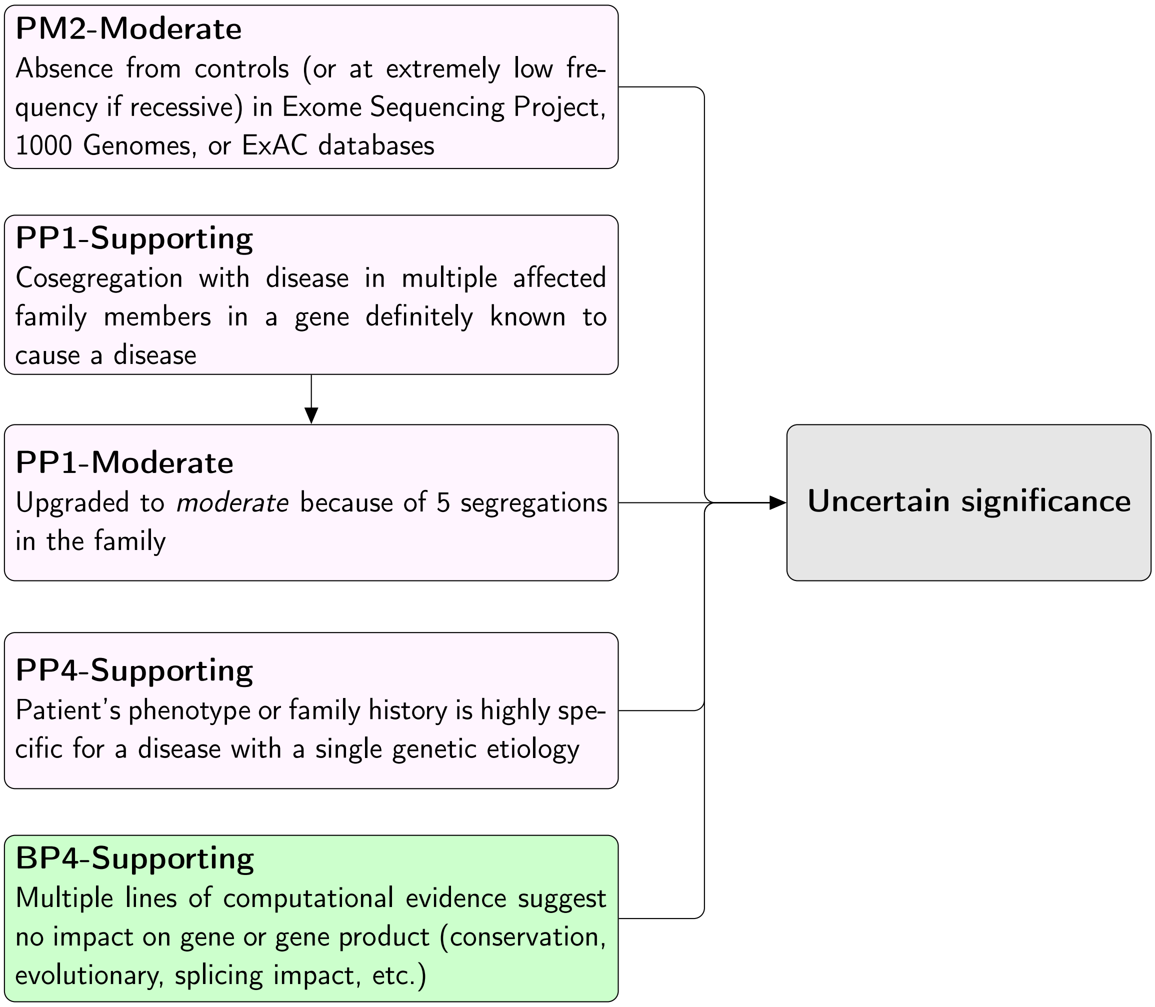


**Figure S6.** *Continued.*

**Table S1.** Rare deleterious exonic variants with required genotypes[a]

| Chromosomal position | Ref. allele | Alt. allele | Gene | Variant type | HGVSc/HGVSp[b] | OMIM disorder associated with the gene |
|---|---|---|---|---|---|---|
| chr1:203,060,296 | C | T | *PPFIA4* | missense | NM_001304331.1:c.2663C>T<br>NP_001291260.1:p.(Ser888Leu) | – |
| chr4:152,881,051 | A | G | *ARFIP1* | missense | NM_001025593.2:c.404A>G<br>NP_001020764.1:p.(Asp135Gly) | – |
| chr5:37,324,053 | C | G | *NUP155* | missense | NM_001278312.1:c.2146G>C<br>NP_001265241.1:p.(Gly716Arg) | Atrial fibrillation, familial, 15 [MIM:615770] |
| chr6:138,412,887 | G | A | *HEBP2* | missense | NM_014320.2:c.427G>A<br>NP_055135.1:p.(Asp143Asn) | – |
| chr6:146,672,389 | A | G | *ADGB* | missense | NM_024694.3:c.1009A>G<br>NP_078970.3:p.(Lys337Glu) | – |
| chr6:148,747,440 | A | C | *UST* | missense | NM_005715.2:c.10A>C<br>NP_005706.1:p.(Lys4Gln) | – |
| chr6:152,330,422 | G | A | *SYNE1* | missense | NM_033071.3:c.14050C>T<br>NP_149062.1:p.(Leu4684Phe) | Spinocerebellar ataxia, autosomal recessive, 8 [MIM:610743]; Emery-Dreifuss muscular distrophy 4 [MIM:612998] |
| chr7:4,265,145 | G | A | *SDK1* | missense | NM_001079653.1:c.1864G>A<br>NP_001073121.1:p.(Asp622Asn) | – |
| chr8:17,563,679 | C | G | *SLC7A2* | missense | NM_001008539.3:c.1748C>G<br>NP_001008539.3:p.(Thr583Ser) | – |
| chr9:20,916,903 | A | T | *FOCAD* | missense | NM_017794.4:c.2818A>T<br>NP_060264.4:p.(Met940Leu) | – |
| chr16:70,860,121 | C | T | *HYDIN* | missense | NM_001270974.2:c.12076G>A<br>NP_001257903.1:p.(Ala4026Thr) | Ciliary dyskinesia, primary, 5 [MIM:608647] |
| chr17:80,372,536 | A | G | *RNF213* | missense | NM_001256071.2:c.12553A>G<br>NP_001243000.2:p.(Lys4185Glu) | Moyamoya disease 2 [MIM:607151] |
| chr17:80,372,545 | G | A | *RNF213* | missense | NM_001256071.2:c.12562G>A<br>NP_001243000.2:p.(Ala4188Thr) | Moyamoya disease 2 [MIM:607151] |
| chr17:82,398,241 | T | C | *OGFOD3* | missense | NM_024648.2:c.778A>G<br>NP_078924.1:p.(Met260Val) | – |
| chr18:31,074,842 | T | C | *DSC2* | missense | NM_004949.4:c.1729A>G<br>NP_004940.1:p.(Ile577Val) | Arrhythmogenic right ventricular dysplasia, familial, 11[MIM:610476] |
| chr18:76,496,529 | - | G | *C18orf65* | frameshift insertion | NM_001272093.2:c.337dup<br>NP_001259022.2:p.(Ala113GlyfsTer6) | – |

[a]Father: heterozygous (0/1); mother: homozygous reference (0/0); sister: homozygous reference or heterozygous; proband: heterozygous; cousin: heterozygous.
[b]HGVS coding and protein sequence names

**Table S2.** Sequencing data metrics for the *RNF213* missense variants

| Family member | Identifier | Affected | Missense variant 1 (c.12553A>G; p.(Lys4185Glu)) | | | | Missense variant 2 (c.12562G>A; p.(Ala4188Thr)) | | | |
|---|---|---|---|---|---|---|---|---|---|---|
| | | | Alt. allele fraction | Read depth (×) | Allele depth (Ref/Alt, ×) | Genotype | Alt. allele fraction | Read depth (×) | Allele depth (Ref/Alt, ×) | Genotype |
| Father | II:8 | No | 0.55 | 110 | 50/60 | A/G (0/1) | 0.55 | 126 | 57/69 | G/A (0/1) |
| Mother | II:9 | No | 0 | 78 | 78/0 | A/A (0/0) | 0 | 87 | 87/0 | G/G (0/0) |
| Sister | III:8 | No | 0.47 | 90 | 47/42 | A/G (0/1) | 0.43 | 102 | 58/44 | G/A (0/1) |
| Proband | III:9 | Yes | 0.44 | 97 | 54/43 | A/G (0/1) | 0.45 | 108 | 56/49 | G/A (0/1) |
| Cousin | III:1 | Yes | 0.61 | 106 | 40/65 | A/G (0/1) | 0.64 | 119 | 43/76 | G/A (0/1) |
| Paternal aunt[a] | II:1 | No | – | – | – | A/G (0/1) | – | – | – | G/A (0/1) |
| Paternal aunt[a] | II:3 | No | – | – | – | A/A (0/0) | – | – | – | G/G (0/0) |
| Paternal uncle[a] | II:5 | No | – | – | – | A/G (0/1) | – | – | – | G/A (0/1) |
| Paternal aunt[a] | II:7 | Yes | – | – | – | A/G (0/1) | – | – | – | G/A (0/1) |
| Cousin[a] | III:2 | Yes | – | – | – | A/G (0/1) | – | – | – | G/A (0/1) |
| Cousin[a] | III:3 | No | – | – | – | A/A (0/0) | – | – | – | G/G (0/0) |

[a]Genotype was obtained with Sanger sequencing.

**Table S3.** dbNSFP v4.0b1 (Liu et al., 2011; Dong et al., 2014; Liu et al., 2016) predictions for the *RNF213* (NM_001256071.2) missense variants reported in this study and by Smith and coworkers (Smith et al., 2014)

| | **Variant c.12553A>G** | **Variant c.12554A>C** | **Variant c.12562G>A** |
|---|---|---|---|
| Position | chr17:80,372,536 | chr17:80,372,537 | chr17:80,372,545 |
| Variant consequences | Missense | Missense | Missense |
| cDNA change | c.12553A>G | c.12554A>C | c.12562G>A |
| Protein change | p.(Lys4185Glu) | p.(Lys4185Thr) | p.(Ala4188Thr) |
| PolyPhen-2 HumDiv prediction[a] | Variable (probably damaging, possibly damaging) | Variable (probably damaging, possibly damaging) | Benign |
| PolyPhen-2 HumVar prediction[a] | Variable (possibly damaging, benign) | Variable (probably damaging, possibly damaging) | Benign |
| SIFT prediction[b] | Deleterious | Deleterious | Tolerated |
| SIFT4G prediction[c] | Deleterious | Deleterious | Tolerated |
| LRT prediction[d] | Deleterious | Deleterious | Neutral |
| MutationTaster prediction[e] | Polymorphism | Polymorphism | Polymorphism |
| MutationAssessor prediction[f] | Medium impact | Medium impact | Medium impact |
| FATHMM prediction[g] | Tolerated | Tolerated | Tolerated |
| fathmm-MKL prediction[h] | Deleterious | Deleterious | Neutral |
| PROVEAN prediction[i] | Neutral | Deleterious | Neutral |
| M-CAP prediction[j] | Possibly pathogenic | Possibly pathogenic | Likely benign |
| CADD score[k] | 26.3 | 24.3 | 15.8 |
| MetaSVM prediction[l] | Tolerated | Tolerated | Tolerated |
| MetaLR prediction [l] | Tolerated | Tolerated | Tolerated |
| phyloP30way (mammals) score[m] | 1.312 | 1.312 | 0.224 |
| phyloP100way (vertebrates) score[m] | 5.992 | 2.067 | 1.019 |
| phastCons30way (mammals) score[n] | 0.308 | 0.295 | 0.057 |
| phastCons100way (vertebrates) score[n] | 1 | 1 | 0.001 |
| GERP++ score[o] | 5.29 | 4.21 | 1.9 |
| Allele frequency in public databases: | | | |
| ExAC | Not present | Not present | Not present |
| 1000 Genomes | Not present | Not present | Not present |
| NHLBI ESP | Not present | Not present | Not present |
| gnomAD | Not present | Not present | Not present |
| Presence in dbSNP | Not present | Not present | Not present |
| Reference | This study | Smith et al., 2014 | This study |

[a] Adzhubei et al., 2010
[b] Ng and Henikoff, 2003
[c] Vaser et al., 2016
[d] Chun and Fay, 2009
[e] Schwarz et al., 2010
[f] Reva et al., 2011
[g] Shihab et al., 2013
[h] Shihab et al., 2014
[i] Choi and Chan, 2015
[j] Jagadeesh et al., 2016
[k] The larger the score (Phred-like), the more likely the variant is damaging (Kircher et al., 2014; Rentzsch et al., 2018)
[l] Dong et al., 2014
[m] The larger the score, the more conserved the site (maximum scores 1.312 for phyloP30way and 10.003 for phyloP100way) (Pollard et al., 2010)
[n] The larger the score, the more conserved the site (maximum score 1 for both phastCons30way and phastCons100way (Siepel et al., 2005)
[o] The larger the score, the more conserved the site (maximum score 6.17) (Cooper et al., 2005, 2010)

## References


Adzhubei, I. A., S. Schmidt, L. Peshkin, V. E. Ramensky, A. Gerasimova, P. Bork, A. S. Kondrashov, and S. R. Sunyaev (2010). A method and server for predicting damaging missense mutations. *Nature Methods 7*(4), 248.

Cho, B.-K. and T. Tominaga (2010). *Moyamoya disease update*. Springer Science & Business Media.

Choi, Y. and A. P. Chan (2015). PROVEAN web server: a tool to predict the functional effect of amino acid substitutions and indels. *Bioinformatics 31*(16), 2745–2747.

Chun, S. and J. C. Fay (2009). Identification of deleterious mutations within three human genomes. *Genome Research 19*, 1553–1561.

Cooper, G. M., D. L. Goode, S. B. Ng, A. Sidow, M. J. Bamshad, J. Shendure, and D. A. Nickerson (2010). Single-nucleotide evolutionary constraint scores highlight disease-causing mutations. *Nature Methods 7*(4), 250.

Cooper, G. M., E. A. Stone, G. Asimenos, E. D. Green, S. Batzoglou, and A. Sidow (2005). Distribution and intensity of constraint in mammalian genomic sequence. *Genome Research 15*(7), 901–913.

DePristo, M. A., E. Banks, R. Poplin, K. V. Garimella, J. R. Maguire, C. Hartl, A. A. Philippakis, G. Del Angel, M. A. Rivas, M. Hanna, et al. (2011). A framework for variation discovery and genotyping using next-generation DNA sequencing data. *Nature Genetics 43*(5), 491–498.

Dong, C., P. Wei, X. Jian, R. Gibbs, E. Boerwinkle, K. Wang, and X. Liu (2014). Comparison and integration of deleteriousness prediction methods for nonsynonymous snvs in whole exome sequencing studies. *Human Molecular Genetics 24*(8), 2125–2137.

Fukui, M. (1997). Guidelines for the diagnosis and treatment of spontaneous occlusion of the circle of Willis ('moyamoya'disease). Research Committee on Spontaneous Occlusion of the Circle of Willis (Moyamoya Disease) of the Ministry of Health and Welfare, Japan. *Clinical Neurology & Neurosurgery 99*, S238–40.

Gelb, B. D., H. Cavé, M. W. Dillon, K. W. Gripp, J. A. Lee, H. Mason-Suares, K. A. Rauen, B. Williams, M. Zenker, and L. M. Vincent (2018). ClinGen's RASopathy Expert Panel consensus methods for variant interpretation. *Genetics in Medicine 20*, 1334–1345.

Hashimoto, N., T. Tominaga, S. Miyamoto, I. Nagata, K. Houkin, N. Suzuki, A. Koizumi, S. Nogawa, J. Nakagawara, K. Kitagawa, et al. (2012). Guidelines for diagnosis and treatment of moyamoya disease (spontaneous occlusion of the circle of Willis). *Neurologia Medico-Chirurgica (Tokyo) 52*(5), 245–266.

Jagadeesh, K. A., A. M. Wenger, M. J. Berger, H. Guturu, P. D. Stenson, D. N. Cooper, J. A. Bernstein, and G. Bejerano (2016). M-CAP eliminates a majority of variants of uncertain significance in clinical exomes at high sensitivity. *Nature Genetics 48*(12), 1581.

Kamada, F., Y. Aoki, A. Narisawa, Y. Abe, S. Komatsuzaki, A. Kikuchi, J. Kanno, T. Niihori, M. Ono, N. Ishii, et al. (2011). A genome-wide association study identifies *RNF213* as the first Moyamoya disease gene. *Journal of Human Genetics 56*(1), 34–40.

Kircher, M., D. M. Witten, P. Jain, B. J. O'Roak, G. M. Cooper, and J. Shendure (2014). A general framework for estimating the relative pathogenicity of human genetic variants. *Nature Genetics 46*(3), 310.

Koizumi, A., K. Nagata, K. Houkin, T. Tominaga, S. Miyamoto, S. Kure, E. Tournier-Lasserve, et al. (2017). *Moyamoya disease explored through RNF213.* Springer.

Lek, M., K. J. Karczewski, E. V. Minikel, K. E. Samocha, E. Banks, T. Fennell, A. H. O'Donnell-Luria, J. S. Ware, A. J. Hill, B. B. Cummings, et al. (2016). Analysis of protein-coding genetic variation in 60,706 humans. *Nature 536*(7616), 285–291.

Li, H. and R. Durbin (2010). Fast and accurate long-read alignment with Burrows–Wheeler transform. *Bioinformatics 26*(5), 589–595.

Liu, W., D. Morito, S. Takashima, Y. Mineharu, H. Kobayashi, T. Hitomi, H. Hashikata, N. Matsuura, S. Yamazaki, A. Toyoda, et al. (2011). Identification of *RNF213* as a susceptibility gene for moyamoya disease and its possible role in vascular development. *PloS One 6*(7), e22542.

Liu, X., X. Jian, and E. Boerwinkle (2011). dbNSFP: a lightweight database of human nonsynonymous SNPs and their functional predictions. *Human Mutation 32*(8), 894–899.

Liu, X., C. Wu, C. Li, and E. Boerwinkle (2016). dbNSFP v3.0: A one-stop database of functional predictions and annotations for human nonsynonymous and splice-site SNVs. *Human Mutation 37*(3), 235–241.

1000 Genomes Project Consortium et al. (2015). A global reference for human genetic variation. *Nature 526*(7571), 68–74.

McKenna, A., M. Hanna, E. Banks, A. Sivachenko, K. Cibulskis, A. Kernytsky, K. Garimella, D. Altshuler, S. Gabriel, M. Daly, et al. (2010). The Genome Analysis Toolkit: a MapReduce framework for analyzing next-generation DNA sequencing data. *Genome Research 20*, 1297–1303.

Mineharu, Y., W. Liu, K. Inoue, N. Matsuura, S. Inoue, K. Takenaka, H. Ikeda, K. Houkin, Y. Takagi, K. Kikuta, et al. (2008). Autosomal dominant moyamoya disease maps to chromosome 17q25.3. *Neurology 70*(24 Part 2), 2357–2363.

Mineharu, Y., K. Takenaka, H. Yamakawa, K. Inoue, H. Ikeda, K. Kikuta, Y. Takagi, K. Nozaki, N. Hashimoto, and A. Koizumi (2006). Inheritance pattern of familial Moyamoya disease: autosomal dominant mode and genomic imprinting. *Journal of Neurology, Neurosurgery & Psychiatry 77*(9), 1025–1029.

Ng, P. C. and S. Henikoff (2003). SIFT: Predicting amino acid changes that affect protein function. *Nucleic Acids Research 31*(13), 3812–3814.

Patel, R. Y., N. Shah, A. R. Jackson, R. Ghosh, P. Pawliczek, S. Paithankar, A. Baker, K. Riehle, H. Chen, S. Milosavljevic, et al. (2017). ClinGen Pathogenicity Calculator: a configurable system for assessing pathogenicity of genetic variants. *Genome Medicine 9*(1), 3.

Pollard, K. S., M. J. Hubisz, K. R. Rosenbloom, and A. Siepel (2010). Detection of nonneutral substitution rates on mammalian phylogenies. *Genome Research 20*(1), 110–121.

Rentzsch, P., D. Witten, G. M. Cooper, J. Shendure, and M. Kircher (2018). CADD: predicting the deleteriousness of variants throughout the human genome. *Nucleic Acids Research 47*(D1), D886–D894.

Reva, B., Y. Antipin, and C. Sander (2011). Predicting the functional impact of protein mutations: application to cancer genomics. *Nucleic Acids Research 39*(17), e118–e118.

Richards, S., N. Aziz, S. Bale, D. Bick, S. Das, J. Gastier-Foster, W. W. Grody, M. Hegde, E. Lyon, E. Spector, et al. (2015). Standards and guidelines for the interpretation of sequence variants: a joint consensus recommendation of the American College of Medical Genetics and Genomics and the Association for Molecular Pathology. *Genetics in Medicine 17*(5), 405.

Schwarz, J. M., C. Rödelsperger, M. Schuelke, and D. Seelow (2010). MutationTaster evaluates disease-causing potential of sequence alterations. *Nature Methods 7*(8), 575.

Shihab, H. A., J. Gough, D. N. Cooper, P. D. Stenson, G. L. Barker, K. J. Edwards, I. N. Day, and T. R. Gaunt (2013). Predicting the functional, molecular, and phenotypic consequences of amino acid substitutions using hidden markov models. *Human Mutation 34*(1), 57–65.

Shihab, H. A., J. Gough, M. Mort, D. N. Cooper, I. N. Day, and T. R. Gaunt (2014). Ranking non-synonymous single nucleotide polymorphisms based on disease concepts. *Human Genomics 8*(1), 11.

Siepel, A., G. Bejerano, J. S. Pedersen, A. S. Hinrichs, M. Hou, K. Rosenbloom, H. Clawson, J. Spieth, L. W. Hillier, S. Richards, et al. (2005). Evolutionarily conserved elements in vertebrate, insect, worm, and yeast genomes. *Genome Research 15*(8), 1034–1050.

Smith, K. R., R. J. Leventer, M. T. Mackay, K. Pope, G. Gillies, M. B. Delatycki, D. J. Amor, M. Bahlo, and P. J. Lockhart (2014). Identification of a novel *RNF213* variant in a family with heterogeneous intracerebral vasculopathy. *International Journal of Stroke 9*(6), E26–E27.

Tarasov, A., A. J. Vilella, E. Cuppen, I. J. Nijman, and P. Prins (2015). Sambamba: fast processing of NGS alignment formats. *Bioinformatics 31*(12), 2032–2034.

Van der Auwera, G. A., M. O. Carneiro, C. Hartl, R. Poplin, G. Del Angel, A. Levy-Moonshine, T. Jordan, K. Shakir, D. Roazen, J. Thibault, et al. (2013). From FastQ data to high-confidence variant calls: the genome analysis toolkit best practices pipeline. *Current Protocols in Bioinformatics 43*(1), 11–10.

Vaser, R., S. Adusumalli, S. N. Leng, M. Sikic, and P. C. Ng (2016). SIFT missense predictions for genomes. *Nature Protocols 11*(1), 1.

Wang, K., M. Li, and H. Hakonarson (2010). ANNOVAR: functional annotation of genetic variants from high-throughput sequencing data. *Nucleic Acids Research 38*(16), e164–e164.
